# Impact of Exposure to Pyraclostrobin and to a Pyraclostrobin/Boscalid Mixture on the Mitochondrial Function of Human Hepatocytes

**DOI:** 10.3390/molecules28207013

**Published:** 2023-10-10

**Authors:** Mélina Carbone, Barbara Mathieu, Yasmine Vandensande, Bernard Gallez

**Affiliations:** Biomedical Magnetic Resonance Research Group, Louvain Drug Research Institute, Université Catholique de Louvain (UCLouvain), Avenue Mounier 73.08, B-1200 Brussels, Belgium; melina.carbone@student.uclouvain.be (M.C.); barbara.mathieu@uclouvain.be (B.M.); yasmine.vandensande@student.uclouvain.be (Y.V.)

**Keywords:** EPR, ESR, fungicides, mitochondrial function, oxygen consumption rate, superoxide, strobilurins, SDHI

## Abstract

Fungicides are widely used in agriculture for crop protection. Succinate dehydrogenase inhibitors (SDHIs) and strobilurins inhibit mitochondria electron transport chain (ETC) in fungi, by blocking complex II and complex III, respectively. Questions regarding their selectivity of action for fungi have been raised in the literature, and we previously showed that boscalid and bixafen (SDHIs) alter the mitochondrial function of human hepatocytes. Here, we analyzed the impact of the exposure of human hepatocytes to pyraclostrobin, a fungicide belonging to the class of strobilurins. Using electron paramagnetic resonance (EPR), we observed a decrease in oxygen consumption rate (OCR) and an increase in mitochondrial superoxide levels after 24 h exposure to 0.5 µM concentration. As a consequence, the content in ATP amount in the cells was reduced, the ratio reduced/oxidized glutathione was decreased, and a decrease in cell viability was observed using three different assays (PrestoBlue, crystal violet, and annexin V assays). In addition, as SDHIs and strobilurins are commonly associated in commercial preparations, we evaluated a potential “cocktail” toxic effect. We selected low concentrations of boscalid (0.5 µM) and pyraclostrobin (0.25 µM) that did not induce a mitochondrial dysfunction in liver cells when used separately. In sharp contrast, when both compounds were used in combination at the same concentration, we observed a decrease in OCR, an increase in mitochondrial superoxide production, a decrease in the ratio reduced/oxidized glutathione, and a decrease in cell viability in three different assays.

## 1. Introduction

**Figure 1:**
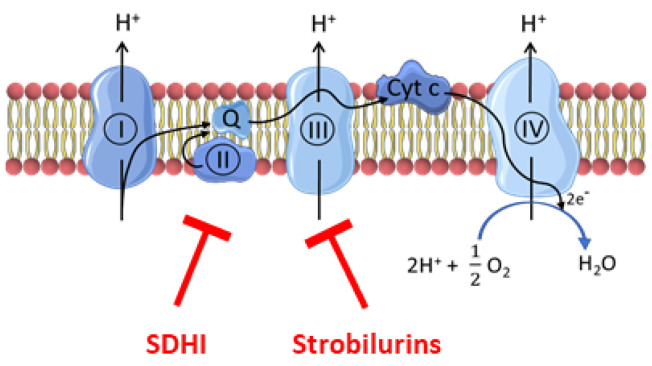
Site of action of fungicides (SDHIs and strobilurins) on the electron transport chain. SDHIs inhibit complex II while strobilurins inhibit complex III.

Plant parasitic fungi have a major socio-economic impact on crops and the food chain. Phytopathogenic fungi are responsible for up to 20% loss of global crop yield [1]. Nowadays, fungicides are extensively used in agriculture for crop protection. The most commonly used classes of fungicides are inhibitors of the electron transport chain (ETC) of fungi, including the succinate dehydrogenase inhibitors (SDHIs) that inhibit complex II and a series of compounds (including strobilurins) that inhibit complex III [2,3,4] (Figure 1).

While crop protection is essential for food chain preservation, there are increasing concerns within the population and public health authorities regarding the selectivity of action of fungicides targeting respiration [5,6]. In the seventies, it was already established that carboxin (5,6-dihydro-2-methyl-1,4-oxathiin-3-carboxanilide) and structurally related oxathin derivatives used as fungicides were also active on beef heart succinate dehydrogenase [7,8,9,10]. Several toxicological studies have shown that some fungicides active on fungi were also altering the mitochondrial function in zebrafish used as model [11,12,13,14,15,16,17]. Surprisingly, a very limited number of studies have been carried out to consider the effect of these substances on human cells [5,18,19,20,21,22]. P. Benit et al. established that the human enzyme SDH (as well as honeybee, earthworm, and fungal SDHs) were all sensitive to the eight SDHI fungicides tested [5]. D. d’Hose et al. tested the mitochondrial function of human cells after exposure for a short time to boscalid and bixafen, the two most used SDHIs [22]. In this study, they measured the oxygen consumption rate (OCR) and the level of mitochondrial superoxide radical using electron paramagnetic resonance (EPR) spectroscopy [22]. The level of mitochondrial superoxide was measured because it is known that an alteration of the mitochondrial function may increase the leakage of free electrons and their reaction with molecular oxygen leading to excessive production of radical superoxide ions. The OCR was significantly decreased in three cell lines after exposure to both SDHIs. They also found that the level of mitochondrial superoxide in HepG2 cells was increased after exposure to bixafen and boscalid leading to an increase in the number of apoptotic cells [22].

**Figure 2:**
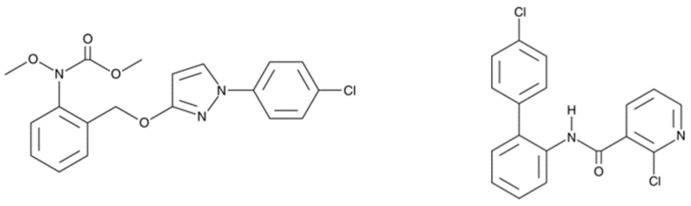
Fungicides analyzed in the present study for their effect on the mitochondrial function of human hepatocytes. **Left**: pyraclostrobin, a strobilurin. **Right**: boscalid, a SDHI.

As strobilurins are also interfering with the ETC, we hypothesized that these compounds may interfere with the mitochondrial function of human cells in a similar way as SDHIs. Therefore, in the present study, we analyzed the impact of the exposure of human hepatocytes to pyraclostrobin (methyl *N*-[2-[[1-(4-chlorophenyl)pyrazol-3-yl]oxymethyl]phenyl]-*N*-methoxycarbamate), one of the most used strobilurins. The HepG2 human cell line was selected because it is a model classically used to assess the hepatoxicity of chemicals or drugs [23,24,25]. The focus on hepatocytes is also justified because, after oral administration of [^14^C]-pyraclostrobin, tissue distribution determination revealed highest amounts of radioactivity in the gastrointestinal tract followed by the liver [26]. Electron paramagnetic resonance (EPR) spectroscopy was selected as a method to assess both the OCR [27,28,29] and the production of mitochondrial superoxide radical [28,29,30,31]. We also evaluated the consequences of the exposure to pyraclostrobin on ATP level as a marker of energetic metabolism, the ratio between reduced and oxidized glutathione as a marker of oxidative stress, and the levels of apoptotic cells. Importantly, as pyraclostrobin and boscalid (2-chloro-*N*-[2-(4-chlorophenyl)phenyl]pyridine-3-carboxamide) (Figure 2) are often associated in commercial preparations (i.e., Pristine^®^ (BASF, Mississauga, ON, Canada), Signum^®^ (BASF, Ecully, France), Visma^®^ (PI Industries Ltd., Gurgaon, India), Sanifol^®^ (Point Andina, Lima, Peru), Pulito^®^ (HD Agro Chemicals, Himachal Pradesh, India), Boscapyr^®^ (Capeagro, Lima, Peru), Bellis^®^ (BASF, Ecully, France), and others), we also evaluated the effect of exposure of human hepatocytes to a mixture of these compounds.

## 2. Results

### 2.1. Pyraclostrobin Induces an Impairment of the Mitochondrial Function of Human Hepatocytes

**Figure 3:**
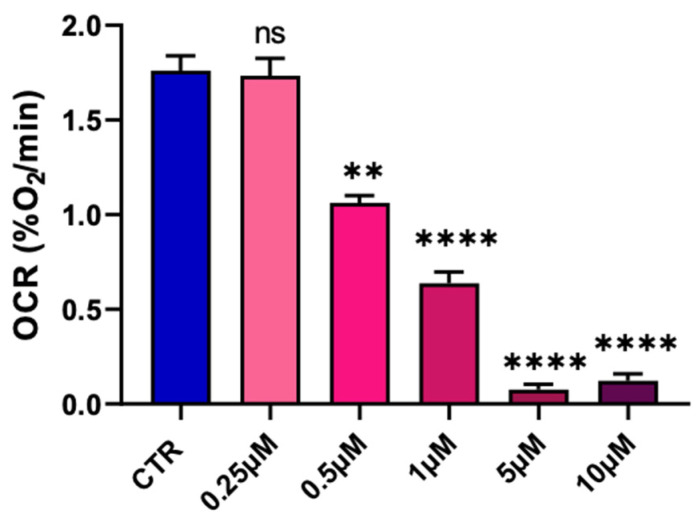
Dose–effect relationship of pyraclostrobin on the oxygen consumption rate established by EPR oximetry. Bars represent mean ± SEM. N = 3, (**): *p* < 0.01, (****): *p* < 0.0001, ns: non significant.

We first screened a potential effect of pyraclostrobin on the oxygen consumption of the hepatocytes. We used EPR oximetry for measuring the OCR by the hepatocytes in sealed capillaries in the presence of a paramagnetic oxygen sensor. Several concentrations of pyraclostrobin were used to determine a possible dose–effect relationship on the OCR (Figure 3). We observed a significant decrease in OCR compared to unexposed cells for concentrations equal to or greater than 0.5 µM.

**Figure 4:**
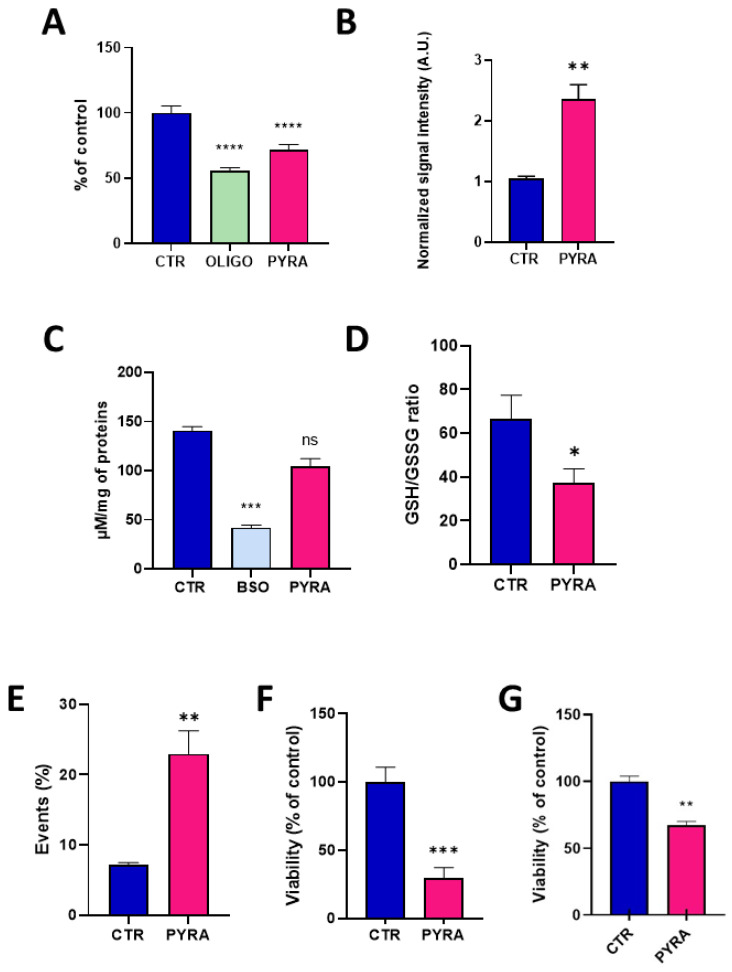
Impact of 24 h exposure to pyraclostrobin 0.5 µM on HepG2 cells. CTR = control, OLIGO = oligomycin, BSO = buthionine-sulfoximine, PYRA = pyraclostrobin 0.5 µM. (**A**) Level of ATP, N = 4; (**B**) level of mitochondrial superoxide radical, N = 3; (**C**) level of total glutathione (reduced GSH and oxidized GSSG forms), N = 5; (**D**) ratio between the levels of reduced GSH and oxidized GSSG forms, N = 5; (**E**) level of early apoptotic cells assessed by flow cytometry, N = 6; (**F**) cell viability assessed by the crystal violet assay, N = 3; (**G**) viability assessed by the PrestoBlue mitochondrial function assay, N = 4. Bars represent mean ± SEM. (*): *p* < 0.05, (**): *p* < 0.01, (***): *p* < 0.001, (****): *p* < 0.0001, ns: non significant.

The concentration of 0.5 µM was used to further explore the consequence of this alteration in mitochondrial function. After 24 h exposure to pyraclostrobin, the level of ATP was significantly decreased (*p* < 0.0001) compared to unexposed hepatocytes, indicating that the energetic metabolism was altered (Figure 4A). In this experiment, we also used oligomycin, an ATPase inhibitor, as a positive control. The level of mitochondrial superoxide radical was evaluated via EPR using the cyclic hydroxylamine MitoTEMPO-H as a marker of the production of mitochondrial reactive oxygen species (ROS). The combined use of pegylated superoxide dismutase (PEG-SOD2) allowed to selectively assign the contribution of mitochondrial superoxide to the oxidation of the probe [28,29]. After 24 h exposure, pyraclostrobin induced a significant increase (*p* < 0.01) in the level of mitochondrial superoxide (Figure 4B). To assess the relevance of this increase in the production of ROS, we evaluated the consequence of a 24 h exposure to pyraclostrobin 0.5 µM on defenses against oxidative stress. We first measured the level of total glutathione (reduced GSH and oxidized GSSG forms). We also used as a positive control buthionine-sulfoximine (BSO), an irreversible inhibitor of γ-glutamylcysteine synthetase known to deplete cellular glutathione levels. Contrarily to BSO, the exposure to pyraclostrobin did not significantly alter the level of total glutathione (Figure 4C). However, a significant part of GSH was consumed by the ROS produced as the ratio GSH/GSSG was significantly reduced after exposure (*p* < 0.05, Figure 4D). Finally, cell viability was assessed through three different assays. Flow cytometry revealed a significant increase (*p* < 0.01) in early apoptotic cells in HepG2 cells exposed to pyraclostrobin for 24 h (Figure 4E). The crystal violet assay (Figure 4F) and the PrestoBlue metabolic assay also revealed a significant decrease in cell viability (Figure 4G).

### 2.2. Mixing Pyraclostrobin and Boscalid Potentiates a Mitochondrial Dysfunction in Human Hepatocytes

**Figure 5:**
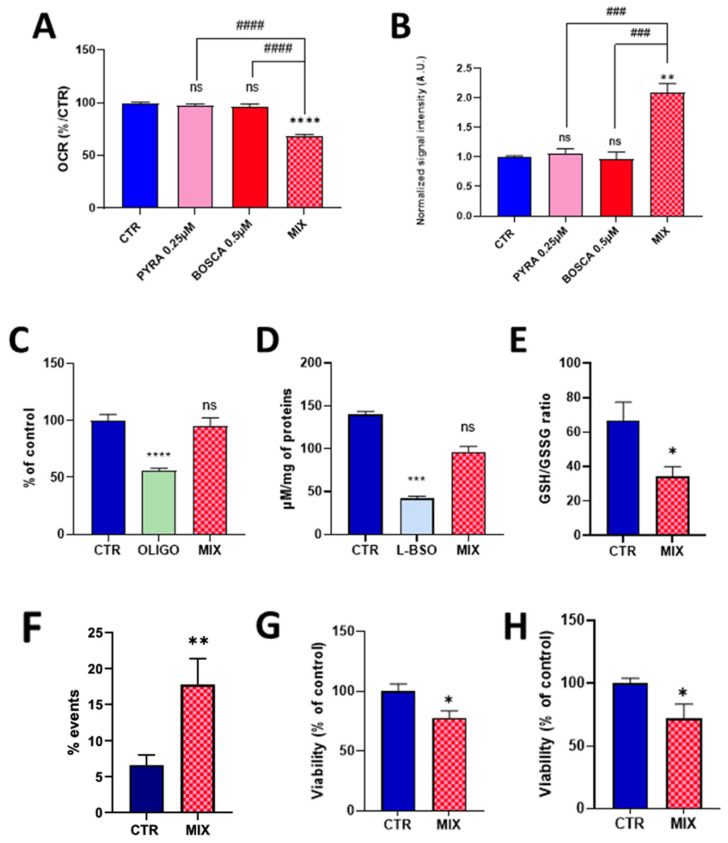
Effect of the exposure of HepG2 cells to a pyraclostrobin 0.25 µM/boscalid 0.5 µM mixture. CTR = control, OLIGO = oligomycin, BSO = buthionine-sulfoximine, BOSCA 0.5 µM = boscalid 0.5 µM, PYRA 0.25 µM = pyraclostrobin 0.25 µM, MIX = pyraclostrobin 0.25 µM/boscalid 0.5 µM mixture. (**A**) OCR (as % of control), N = 3; (**B**) level of mitochondrial superoxide radical (compared to control), N= 3; (**C**) ATP level (compared to control), N = 4; (**D**) level of total glutathione (reduced GSH and oxidized GSSG forms); (**E**) ratio between the levels of reduced GSH and oxidized GSSG forms, N = 5; (**F**) level of early apoptotic cells assessed by flow cytometry, N = 6; (**G**) cell viability assessed by the crystal violet assay, N = 3; (**H**) viability assessed by the PrestoBlue mitochondrial function assay, N = 4. Bars represent mean ± SEM. Symbols used for the comparison with the control: (*): *p* < 0.05, (**): *p* < 0.01, (***): *p* < 0.001, (****): *p* < 0.0001, ns: not significant. Comparison between exposure to individual fungicide and mixture of fungicides: (###): *p* < 0.001, (####): *p* < 0.0001.

As pyraclostrobin is often associated with the SDH inhibitor boscalid in commercial preparations, and as it was previously demonstrated that boscalid induced a mitochondrial dysfunction in HepG2 cells [22], we evaluated the effect of exposure of human hepatocytes to a mixture of these compounds. To potentially evaluate if a synergy exists between the two compounds, we first selected concentrations at which neither compound induced an alteration of the mitochondrial function when used separately. At the concentration of 0.25 µM for pyraclostrobin and 0.5 µM for boscalid, we did not observe any significant effect on OCR (Figure 5A) or on mitochondrial superoxide production (Figure 5B).

In sharp contrast, the association of both compounds at the same concentration led to a significant decrease in OCR (Figure 5A) and a significant increase in the production of mitochondrial superoxide (Figure 5B). Looking to the possible consequences of this mitochondrial function alteration, we did not observe a significant impact of energetic metabolism as the level of ATP was not significantly different between exposed and non-exposed cells to the pyraclostrobin/boscalid mixture (Figure 5C). While the exposure to the mixture did not alter the level of total glutathione (Figure 5D), we observed a significant decrease (*p* < 0.05) in the ratio GSH/GSSG (Figure 5E). Finally, we observed a significant increase in the % of apoptotic cells compared to the unexposed cells as measured by flow cytometry (Figure 5F). Both the crystal violet assay (Figure 5G) and the PrestoBlue assay (Figure 5H) also showed a significant decrease (*p* < 0.05) in viability.

## 3. Discussion

The alteration of mitochondrial function (as revealed by the decrease in oxygen consumption, Figure 3) observed after 24 h of exposure to pyraclostrobin (at a concentration as low as 0.5 µM) induced important cellular consequences. First, it modified the energetic metabolism as the level of ATP, the energy store in biological systems, was significantly decreased (Figure 4A). Another important consequence relied in the increase in mitochondrial superoxide (Figure 4B) due to the electron leak from the ETC. This resulted in a decrease in cellular antioxidant capacity, as demonstrated by the decrease in the ratio GSH/GSSG (Figure 4D). In addition, the cell viability was decreased as revealed by three independent assays. The PrestoBlue assay is actually not directly measuring cell survival, but is rather a measurement of the mitochondrial activity. The apparent decrease observed in cell viability could therefore be the result of a decrease in mitochondrial activity (Figure 4G), a result consistent with the decrease in OCR. The crystal violet assay, that quantifies DNA (and therefore the number of cells) thanks to this intercalating dye, also revealed a decrease in cell viability (Figure 4F). In addition, flow cytometry with annexin V revealed a significant increase in the number of early apoptotic cells (Figure 4E). Overall, our results are consistent with previous studies that used pyraclostrobin on other models showing the absence of selectivity of pyraclostrobin for fungi [15,16,21]. The results obtained for the association pyraclostrobin/boscalid were even more troublesome as there was a clear potentiation of the effect on the mitochondrial function of the hepatocytes exposed to this mixture (Figure 5). While the low concentration of pyraclostrobin (0.25 µM) and boscalid (0.5 µM) had no effect when used separately, the combined exposure led to a decrease in oxygen consumption, and an increase in superoxide production with a consequent decrease in defense against oxidative stress and alteration in cell viability.

An open question is to know if our results obtained with pyraclostrobin are relevant for the health safety of the farmers or public exposed to this fungicide. As already discussed in our previous publication on SDHIs [22], there is a large uncertainty regarding the real concentrations achieved in the blood and the tissues of people potentially exposed to fungicides, whatever these fungicides are. The concentrations achieved for exposed farmers will depend on the frequency of spreading and exposure periods, the amounts of fungicides manipulated, and the means of protection used. For the general public, it will depend on the residence area (urban or rural, and proximity to spread areas) and potentially contaminated food diets. Public health authorities have defined AOELs (acceptable operator exposure levels), ADI (acceptable daily intake) for each fungicide, and MRLs (maximal residue levels) for each fungicide and each type of crop [32]. For example, the European Food Safety Authority (EFSA) provides an annual report which examines pesticide residue levels in foods on the European market [33]. AOELs and ADIs are actually defined by extrapolation of toxicology data obtained in animals and by defining a theoretical security factor. For pyraclostrobin, the AOEL and ADI are 0.015 mg/kg/day and 0.03 mg/kg/day, respectively [34]. Considering an oral absorption of 50% [34], for a person of 70 kg body weight exposed to a single dose close to the ADI (2.1 mg or 5.42 µmol), considering a volume of blood of 5 L, the peak of concentration in the blood would achieve a value around 0.5 µM. The elimination of pyraclostrobin is characterized by two phases with long half-lives of 9 h and 37 h, respectively [35]. For boscalid, the AOEL and ADI are 0.1 mg/kg/day and 0.04 mg/kg/day, respectively [36]. Applying the same reasoning for boscalid, considering an oral absorption of about 50%, the peak of concentration in the blood would achieve a value around 0.8 µM if exposed at a dose close to the ADI. The elimination of boscalid is characterized by two phases with long half-lives of 7 h and 41 h, respectively [37]. Importantly, the liver is the organ that accumulates the most of both fungicides. Based on the theoretical considerations mentioned above, we can see that the concentrations used in our study (0.5 µM pyraclostrobin used alone, or 0.25 µM pyraclostrobin/0.5 µM boscalid used in the mixture) are close to the concentrations likely achieved after a single exposure that is considered as being acceptable according to the ADI. Even if the compounds are cleared from the body, the long half-lives of elimination suggest the possibility of having prolonged exposure. In addition, we cannot exclude that metabolites with structures close to the one of the active ingredients could also act on the mitochondrial function of hepatocytes. Of note, it would be extremely difficult to use appropriate models for predicting both concentrations/toxic effects in mixtures knowing that commercial preparations contain their proper concentrations of compounds in different proportions and acknowledging the fact that each active molecule possesses its own pharmacokinetics (absorption, distribution, metabolism, and elimination).

The present work contributes to the important debate on the use of fungicides targeting mitochondrial respiration. On the one hand, the agrochemical industry promotes and justifies the use of pesticides to sustain crop productivity, to insure crop protection, and to minimize the probability for the food for being contaminated by dangerous mycotoxins. Left untreated, fungal diseases could reduce the yield by an average of around 20% [38]. Many important pathogens are very effectively controlled by SDHIs and strobilurins. The formulation of multi-active ingredients could lead to an increase in efficiency for farmers, by reducing the number of required applications, saving both money and time. In addition, by targeting different sites, the use of cocktails of fungicides could theoretically minimize the risk of emergence of resistance by comparison with the use of a single agent. On the other hand, the use of non-selective compounds generates important concerns regarding the pollution of the environment, negative biodiversity impact, and, last but not least, toxicity for humans. Our in vitro study demonstrates that a short-time exposure to pyraclostribin induced a mitochondrial dysfunction in human cells. More importantly, the “cocktail effect” that we demonstrated in the present study provides alarming signs regarding a potentiation of toxic effects.

The lessons from these in vitro experiments should be carefully considered before any extrapolation to human health. It is beyond the scope of this experimental study to provide strong statements and definitive recommendations regarding the use or discontinuation of products. However, we believe that the results obtained should stimulate further research in different areas. Here, we focused on a single exposure. In the real world, it is likely that the public or operators/farmers would be exposed chronically. Future studies should include repeated exposure to address their potential consequences. Other potentially exposed cells (i.e., from the skin, lungs, and kidneys) should be included to assess the potential relevance of the present findings. In addition, biomonitoring studies are crucial in the future to evaluate the real level of exposure for people in daily life or after incidents in the handling of fungicide preparations [39]. Finally, special attention should be given to associations of fungicides. It is essential in the future to address the possible detrimental consequences of combined exposures, even if it would be very difficult to analyze all possible combinations of existing fungicides. Finally, even if it is beyond the scope of the sole focus on fungicides, future studies should also consider the possible effects of a combined exposure to fungicides and drugs. Knowing that the exposure to fungicides in this study altered the level of available glutathione in hepatocytes and that GSH protects against the toxicity of many drugs (for example, acetaminophen, to cite one of the most used pain-relieving drugs [40]), it would be interesting to further consider if fungicides could have a potential impact on the toxicity of drugs.

## 4. Materials and Methods

### 4.1. Reagents

Pyraclostrobin PESTANAL^®^ (CAS number: 175013-18-0) and Boscalid PESTANAL^®^ (CAS number: 188425-85-6) were purchased from Supelco (Sigma-Aldrich, Hoeilaert, Belgium) and dissolved in DMSO (Sigma-Aldrich). Oligomycin (CAS number: 1404-19-9) and L-buthionine sulfoximine (L-BSO, CAS number: 83730-53-4) were acquired from Sigma-Aldrich (Hoeilaert, Belgium).

### 4.2. Cell Culture

HepG2 cells were purchased from The American Type Culture Collection (ATCC) (Manassas, VA, USA) and cultured in Minimum Essential Medium (MEM^®^) (Thermo Fisher, Merelbeke, Belgium) with 10% of heat-inactivated fetal bovine serum (FBS) (Sigma-Aldrich). Cells were maintained at 37 °C in humidified atmosphere with 5% CO_2_.

### 4.3. EPR Oximetry

Electron paramagnetic resonance (EPR) can be used to measure variations in oxygen levels in samples and, afterwards, the oxygen consumption rate (OCR) of cells in suspension sealed in a capillary system. A very detailed protocol with all steps of the assay has been published elsewhere [23]. EPR oximetry is based on the use of an oxygen-sensing probe, ^15^N-PDT (4-oxo-2,2,6,6-tetramethylpiperidine-d_16_-^15^N-1-oxyl) (CDN Isotopes, Pointe-Claire, QC, Canada) the linewidth of which is very narrow, providing a high sensitivity to measure the broadening induced by the oxygen content in the preparation. The ^15^N-PDT linewidth was correlated with the % of oxygen present in the sample with a calibration curve. The day prior to the experiment, the cells were treated with fungicides (pyraclostrobin and/or boscalid) or the appropriate control (DMSO). The cells were harvested to form a stock solution of 5 × 10^6^ cells/mL of culture medium. A mixture containing 60 µL of cell suspension, 40 µL of Dextran solution (20%), and 4 µL of ^15^N-PDT (2 mM) was transferred into a hematocrit capillary sealed with gum. The sealed capillary was inserted into a quartz tube and put into the EPR cavity heated at 37 °C with continuous nitrogen flow. The OCR of cells was measured using a Bruker EMX-Plus spectrometer operating in X-band (9.85 GHz) and equipped with a PremiumX ultra low noise microwave bridge and a SHQ high sensitivity resonator. The experimental parameters were: microwave power, 2.518 mW; modulation amplitude, 0.005 mT; modulation frequency, 100 kHz; center field, 335 mT; sweep time, 15 s; and sweep width, 1.5 mT. The measurement started 2 min after having mixed the probe with the cells. Fifteen spectra with a time delay of 1 min were recorded to build the curve of evolution of oxygen concentration as a function of time.

### 4.4. EPR Mitochondrial Superoxide Measurement

To measure mitochondrial superoxide by EPR, Mito-TEMPO-H (2-(2,2,6,6-Tetramethylpiperidin-1-oxyl-4-ylamino)-2-oxoethyl triphenylphosphonium chloride) (Enzo Lifescience, Antwerpen, Belgium), a cyclic hydroxylamine able to detect superoxide in complex biological media, was used. The tryphenylphosphonium moiety (TPP^+^) enables this probe to enter and accumulate within mitochondria, allowing specific measurement of mitochondrial superoxide. A very detailed protocol with all steps of the assay has been published elsewhere [23]. The Mito-TEMPO-H stock solution was flushed with argon prior to and during pipetting to avoid probe oxidation. The day prior to the experiment, the cells were treated with fungicides (pyraclostrobin and/or boscalid) or the appropriate control (DMSO). The cells were harvested to form a stock solution of 15 × 10^6^ cells/mL of culture medium. A mixture containing 37 µL of cell suspension, 0.50 µL of DTPA (100 mM), 5 µL of PBS ((1×)—pH 7.4), and 7.5 µL of Mito-TEMPO-H (1 mM) was transferred into a gas-permeable polytetrafluoroethylene tube (inside diameter 0.025 in., wall thickness 0.002 in.) with a cutting of 12 cm using a needle and folded in 6 before being inserted into an open quartz tube. The quartz tube was inserted in the EPR cavity and heated at 37 °C with continuous air flow. The superoxide production contribution was assessed by making another measurement using the same conditions but adding 2.5 µL of PEG-SOD2 (4000 U/mL) incubated for 15 min before adding the Mito-TEMPO-H probe, allowing the cellular uptake of the enzyme and the intracellular scavenging of superoxide. The superoxide production was monitored using the same system as described for the OCR measurements. The experimental parameters were: microwave power, 20 mW; 100 kHz; modulation amplitude, modulation frequency, 0.1 mT; center field, 336.5 mT; sweep time, 30.48 s; and sweep width, 1.5 mT. The measurement started 2 min after mixing the probe with the cells, counting 11 points with a time delay of 40 s between each point. Data analysis was performed using “Integration & derivative” and “double integration” of the Bruker Xenon Spin fit program on selected regions of the peaks. The final file was saved to extract double integration (DI) data at each timepoint. Point 1 DI was subtracted from point 11 DI for each condition. The superoxide contribution was measured by subtracting the mean PEGSOD2 DI from the mean control DI.

### 4.5. Intracellular ATP Quantification

The intracellular ATP was quantified using the Celltiter-Glo^®^ Luminescent cell viability assay purchased from Promega (Madison, WI, USA). The cells were seeded in a 96-well plate at the density of 7500 cells/well 48 h prior to quantification. Cells were treated for 24 h with fungicides (pyraclostrobin and/or boscalid) or the appropriate control (DMSO). To assess the validation of the test, a positive control using oligomycin, an ATP synthase inhibitor, at 0.1 µg/mL was added. To quantify the intracellular ATP, the cells were washed twice with phosphate-buffered saline (PBS), pH 7.4 (Thermo Scientific, Waltham, MA, USA), and the Celltiter-Glo^®^ Reagent mixed in PBS was added following the manufacturer’s instructions. The luminescence was measured after 5 min of incubation using a SpectraMaxE2 plate reader (Molecular Devices, San Jose, CA, USA). The quantity of ATP was assessed from a calibration curve of ATP disodium (SigmaAldrich) from 10 nM to 1 µM. For each condition, the protein quantification was performed using a BCA Protein Assay Kit (PierceTM, Thermo Fisher). The concentration of intracellular ATP was normalized by mg of protein and presented as a percentage of the control condition (DMSO).

### 4.6. Intracellular Glutathione Quantification

Intracellular glutathione levels were assessed using the Glutathione colorimetric detection kit (Invitrogen, Waltham, MA, USA, Thermo Fischer). HepG2 cells were seeded in 100 mm^2^ Petri dishes at the density of 10^6^ cells per dish. Cells were treated for 24 h with fungicides (pyraclostrobin and/or boscalid) or the appropriate control. The validation of the test was assessed by using a positive control, L-Buthionine sulfoximine (L-BSO), a glutathione synthase inhibitor, at the concentration of 25 µM. Cells were washed with PBS and detached by trypsinization. They were centrifuged and resuspended in a solution of 5% 5-sulfo-salicylic acid dihydrate (5-SSA) (CAS number: 5965-83-3) (Sigma-Aldrich) to allow the precipitation of proteins. The total glutathione (GSH_tot_) and the oxidized form (GSSG) were quantified following the manufacturer’s instructions. The absorbance was measured with a SpectraMaxE2 plate reader (Molecular Devices) at the wavelength of 405 nm. The reduced form (GSH) was deduced via the subtraction of GSH_tot_ from the GSSG concentration. The proteins were quantified for each condition using a BCA Protein Assay kit (PierceTM, Thermo Fisher). The concentration of intracellular glutathione was normalized by mg of protein.

### 4.7. Apoptotic Changes by Flow Cytometry

Apoptotic changes were measured by flow cytometry using eBioscience™ Annexin V Apoptosis Detection Kit APC (Thermo Fischer). The cells were seeded 2 days prior to treatment in T75 flasks. On the day of the experiment, the cells were treated with fungicides (pyraclostrobin or/and boscalid) or the appropriate control (DMSO) for 2 h. The cells were washed twice with Versene solution (0.48 mM of EDTA in PBS) and detached with Trypsin-EDTA 0.05% (Thermo Fischer). They were harvested into their respective old media, centrifuged, and transferred into a FACS tube with fresh medium. The staining protocol was performed following the manufacturer’s instructions. Data were acquired using a BD FACSCantoII flow cytometer equipped with BD FACSDiva v9.0 Software and processed with FlowJo v10.9 software. A total of 10,000 events were measured for each condition and divided into sub-populations according to unstained control cells and distinguished between alive (AnV^−^ PI^−^), early apoptotic (AnV^+^ PI^−^), and late apoptotic/necrotic (AnV^+^ PI^+^).

### 4.8. Cell Viability by PrestoBlue

Cell viability assessment by PrestoBlue is based on the reduction of resazurin by the reducing environment of cells to fluorescent resofurin. This transformation occurs only in living cells and allows the quantification of viability. The cells were seeded in a 96-well plate two days prior to the experiment at the density of 7500 cells/well. They were treated with fungicides (pyraclostrobin or/and boscalid) or the appropriate control (DMSO) for 24 h. A mix of cell medium with 10% PrestoBlue cell viability reagent (Thermo Fischer) was added to each well and incubated for 2 h at 37 °C. Absorbance was measured at 570 nm on a SpectraMaxE2 plate reader (Molecular Devices). Results are presented as a percentage of the control condition (DMSO).

### 4.9. Cell Viability by Crystal Violet

PrestoBlue allows sensitive quantification of viable cells. Nevertheless, it relies on the reducing potential of mitochondria. Since then, impairment of the mitochondrial function could lead to an under/over-estimation of cell viability. To complete the PrestoBlue analysis, crystal violet was performed. Crystal violet stains the nuclear DNA of cells. Cells were seeded 2 days prior to the experiment in a 96-well plate at the seeding density of 7500 cells/well. They were treated identically to the PrestoBlue analysis. On the day of the experiment, the cells were washed twice with PBS and 50 µL of crystal violet solution (1%) (CAS number: 548-62-9) (Sigma-Aldrich) and incubated for 20 min at room temperature on an orbital plate (20 oscillations/min). Then, the cells were washed with PBS to eliminate the excess of the crystal violet solution and the remaining dead cells. The stain dried for 2 h and was resuspended in methanol. The absorbance was measured at 570 nm.

## Data Availability

Rough data are available on request.
